# Experimental realization of spatially separated entanglement with continuous variables using laser pulse trains

**DOI:** 10.1038/srep13029

**Published:** 2015-08-17

**Authors:** Yun Zhang, Ryuhi Okubo, Mayumi Hirano, Yujiro Eto, Takuya Hirano

**Affiliations:** 1 Department of Engineering Science, The University of Electro-Communications, 1-5-1 Chofugaoka, Chofu-shi, Tokyo 182-8585, Japan; 2 Department of Physics, Gakushuin University, 1-5-1 Mejiro, Toshima-ku, Tokyo, 171-8588, Japan

## Abstract

Spatially separated entanglement is demonstrated by interfering two high-repetition squeezed pulse trains. The entanglement correlation of the quadrature amplitudes between individual pulses is interrogated. It is characterized in terms of the sufficient inseparability criterion with an optimum result of 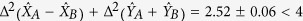 in the frequency domain and 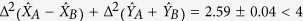 in the time domain. The quantum correlation is also observed when the two measurement stations are separated by a physical distance of 4.5 m, which is sufficiently large to demonstrate the space-like separation, after accounting for the measurement time.

Quantum entanglement is one of the most fundamental properties of quantum mechanics and plays an essential role for demonstrating Einstein, Podolsky, and Rosen (EPR) paradox^1^, revealing a contradiction between local realism and the completeness of quantum mechanics, and providing a path to novel quantum technologies^2^. Of particular importance for application to quantum communications such as quantum cryptography are optical experiments based on correlations in discrete variables or continuous variables^2^,^3^,^4^. In the discrete variable regime, the correlation of spatially separated photon pairs, usually generated by parametric down conversion, has been investigated for each individual measurement^5^,^6^,^7^. On the other hand, the realization of the continuous variable EPR paradox, which was analyzed by Reid^8^ for a realistic situation where there is less than perfect correlation, involved correlations between two complementary quadrature phase amplitudes of two beams. The experimental demonstration of such correlations has been reported using parametric amplification or oscillation with measurements by homodyne detection^9^,^10^,^11^.

A recent review of this field has summarized the relationship of the EPR paradox and Bell’s theorem. The experimental demonstration of the paradox differs from Bell’s inequality in that the former tests the incompatibility of local realism with the completeness of quantum mechanics, whereas the latter tests the incorrectness of local realism : the conclusions of the latter are stronger that of the former^2^. More recently, Wiseman *et al.* rigorously defined the concept of quantum steering^12^ which was introduced by Schrödinger in 1935^13^ and has a close connection to the sufficient correlation of EPR paradox^14^. They proved that steerable states are a strict subset of entangled states, and a strict superset of the states that can violate the Bell’s inequality.

In recent years, there has been an effort to achieve loophole free EPR experiment taking advantage of the progress of experimental techniques^15^,^16^,^17^. For this purpose, it is first necessary that measurements at two sites (for example, Alice’s and Bob’s site) are causally separated. Second, there must exist sufficient quantum correlation between measurements made at Alice and Bob. In this respect, experiments with continuous variables have successfully demonstrated the criterion of sufficient quantum correlation. However causal separation of measurement events remains absent^9^,^10^,^11^. On the other hand, causal separation was completely achieved in experiments with discrete variable^5^,^6^,^7^, but it is a challenging work to close the loophole of sufficient correlation for Bell’s inequality due to the limited efficiency of the photon detectors^18^, even though the loophole free EPR steering has recently been demonstrated in the state-of-the-art experiment^15^. In this context, our research group has addressed the demonstration of loophole free EPR paradox with continuous variable: in particular, we choose quadrature phase amplitudes of each individual pulses as observables. In other words, the criterion of sufficient quantum correlation is being investigated with continuous variables of each individual pulses. And, causal separation of the measurement events will be achieved by taking sufficient physical distance between two measurement stations in consideration of measurement time of each individual pulses.

In this paper, the first step of the program is reported, namely, the time-domain observation of quantum correlations between individual entangled pulses with high repetition rate of 76 MHz and extension of the separation distance between Alice and Bob to achieve the causal separation criterion. This scheme has several advantages compared with previously reported pulsed entanglement experiments^19^,^20^,^21^. The distance satisfying the causal separation is roughly proportional to the reciprocal of laser pulse repetition rate times the speed of light in our approach using pulse-resolved homodyne detectors, in which one measurement of the field quadrature is yielded by one laser pulse and the analysis is directly obtained from these individual pulse measurements. The use of pulse trains with high repetition rate enables to shorten the required distance to a feasible value in the laboratory (4 m in our case). Thus the demonstration of time-domain entanglement with high-repetition-rate pulse trains is a significant step toward observation of the causally separated EPR paradox and, to the best of our knowledge, this is the first demonstration of entanglement that is sufficiently separated to verify the casual separation criterion with continuous variables. In addition, the time-domain observation of entanglement with high repetition rate would yield a benefit to the realization of quantum communication such as entanglement based quantum key distribution.

## Methods

### Experimental setup

**Figure 1:**
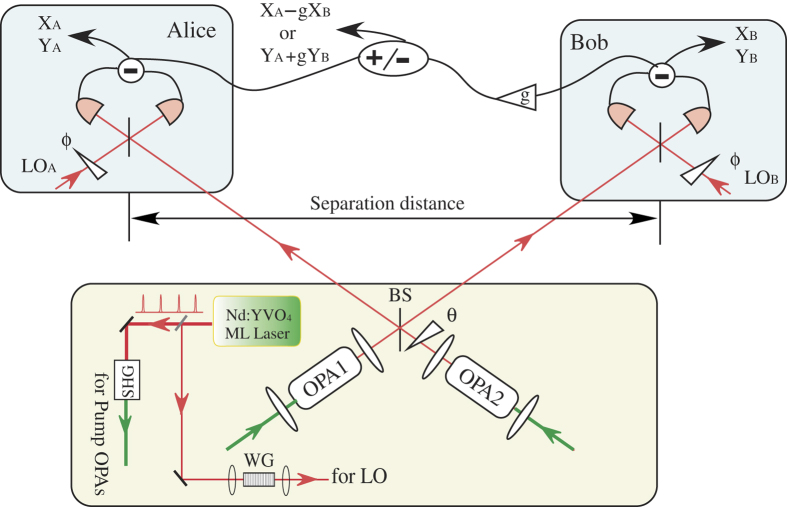
Schematic diagram of the generation of spatially separated entanglement. OPA: optical parametric amplifier; BS: 50/50 beam splitter; LO: local oscillator.

A schematic diagram of our experiment is illustrated in Fig. 1. It is similar to that used for the generation of entanglement using laser pulses^19^. The primary source of the experiment is a cw mode-locked Nd:YVO_4_ (VAN) laser, operating at 1064 nm with a pulse duration of 7 ps and a pulse repetition rate of 76 MHz. Most of laser beam is directed to a second harmonic generator to produce 532 nm light to pump optical parametric amplifiers (OPAs), which are employed to generate quadrature amplitude squeezed pulse trains. It is important to maximize the mode matching between the local oscillator (LO) and the entangled beam. To do this, the LO pulses are obtained from a small portion of the laser emission. They are then passed through a single-mode periodically poled lithium niobate (PPLN) waveguide, which is used as the nonlinear material in the OPAs, to ensure proper spatial mode matching with the signal.

The entanglement is produced by combining two squeezed pulse trains at a 50/50 beam splitter (BS) with a relative phase of *π*/2 and an observing visibility of (98 ± 1)%. Two squeezed pulse trains at 1064 nm are produced from single-pass OPAs pumped by the frequency-doubled laser pulses. Each OPA consists of a PPLN waveguide with a length of 5.0 mm and effective core area of 3 *μ*m × 5 *μ*m as the second-order nonlinear material. Squeezing of about 3.4 ± 0.2 dB below the shot noise limit is obtained over a bandwidth of 200 MHz. Entanglement can be stably observed during the whole measurement. Details of how the squeezed states are generated and measured can be found elsewhere^19^,^22^.

The output beams from the BS are sent to Alice and Bob who are spatially separated, and they are then interrogated in homodyne detectors (HDs) having a bandwidth of 200 MHz by mixing them with local oscillators (LOs). The common path arrangement is used in the interferometers, in which the entangled pulse and the LO collinearly propagated with orthogonal polarization. Due to this arrangement, the relative phase between the entangled pulse and the LO is moderately stabilized. Either the amplitude (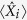, where *i* = *A* or *B*) or the phase 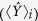 quadrature can be measured by controlling the phase of the LO light. The quadrature operators are defined in terms of field annihilation 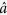 and creation 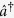 operators, 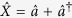 and 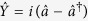. The quantum correlation of entanglement is analyzed both in the frequency domain and in the time domain. In the time domain, the output of the HD is sampled with a digital oscilloscope at sampling rate of 5 G samples/s and an analog bandwidth of 2 GHz. This pulsed homodyning is technically much more challenging than frequency-resolved homodyning. In principle, the repetition frequency signal and its harmonic signals should be completely subtracted by adjusting the balance of homodye detection system. In our homodyne detection system, the repetition frequency signal is reduced more than 40 dB comparing with the level that one of photodiodes is blocked. The residual of repetition frequency signal can be further filtered by employing a notch filter. By blocking the two squeezed states, the detection is shown to be shot-noise limited (SNL), exhibiting a linear dependence on the LO power up to 2 mW, and the SNL is more than 10 dB above the electronic noise of the HD. The overall homodyne detection efficiency is dominated by the quantum efficiency of the photodiodes and by the visibility of the LO and the entangled beams. We observed a visibility of *η*_*V*_ = 0.92 ± 0.03 between each entangled beam and its homodyne LO beam. The quantum efficiencies of the photodiodes are measured to be *η*_*D*_ = 0.85 ± 0.03. Hence the overall detection efficiency is 
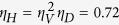
. A total efficiency of propagation from the OPA to the HDs is approximately 0.95. Even when the propagation distance is increased from 20 cm to 4.5 m, no additional losses are increased in the propagation efficiency.

### Independent measurement and inseparability criterion

**Figure 2:**
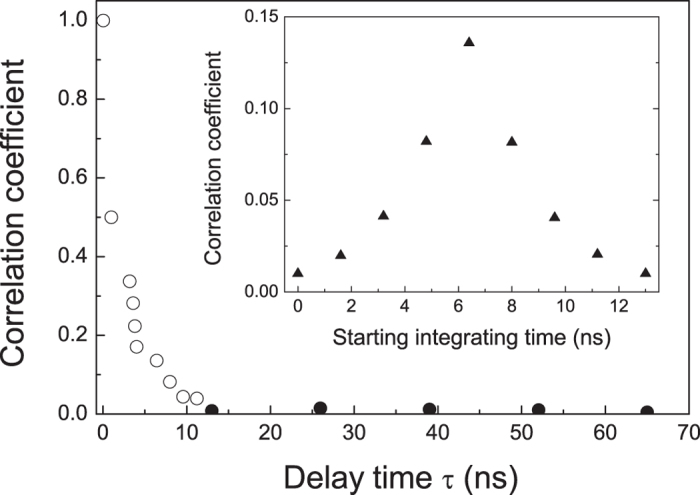
Correlation coefficient between measurements at a given time of “T” and the delays time *τ*. The inset shows the correlation coefficient for measured quadrature sequences of two adjacent pulse trains with different starting integrating time. The power of local oscillator is 1.6 mW.

In the time domain measurement, one quadrature value is sampled for per pulse by integrating HD output over a time interval. Considering the laser pulse repetition rate of 76 MHz (whose reciprocal is 13 ns), an integrating time window of 13 ns is chosen. To achieve the causal separation criterion, each measurement should be independent of the adjacent ones, *i.e.* one measured result is irrelevant to its adjoining measurements. To verify that, we calculated the correlation coefficient between two measured quadrature sequences corresponding to the measured results at a given starting time of “T” and a delay time “*τ*” for a pulse train. Figure 2 gives the obtained correlation coefficient versus the delay time. Each point corresponds to an average value over ten measurements. The obtained correlation coefficient is 0.04 when the delay time is 10 ns, and it is further reduced to below 0.01 at a delay time of 13 ns. The correlation coefficient is also strongly dependent on the starting time “T”. In the inset of Fig. 2, the correlation coefficient is plotted for two calculated quadrature sequences of laser pulse trains with a fixed time delay of *τ* = 13 ns and an integration window of 13 ns for different starting times. Depending on the starting time, a single pulse can be divided into two adjacent integrating windows, in which case the quadrature value is obtained from the two parts of adjacent laser pulses. A maximum correlation coefficient of 0.15 is observed when the starting time is 6.5 ns, corresponding to the center of the integrating window of 13 ns. These results demonstrate individual pulse measurements within a duration of 13 ns. Hence, when the two observers Alice and Bob are spatially separated by more than 4 m, the causal separation criterion will be achieved^2^.

The inseparability criterion for an entanglement is related to their degree of quantum correlation. It is based on the variance of the difference and sum of a pair of canonical conjugates variable and can be written as^23^,^24^


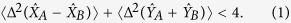


## Results

### Short distance separation

**Figure 3:**
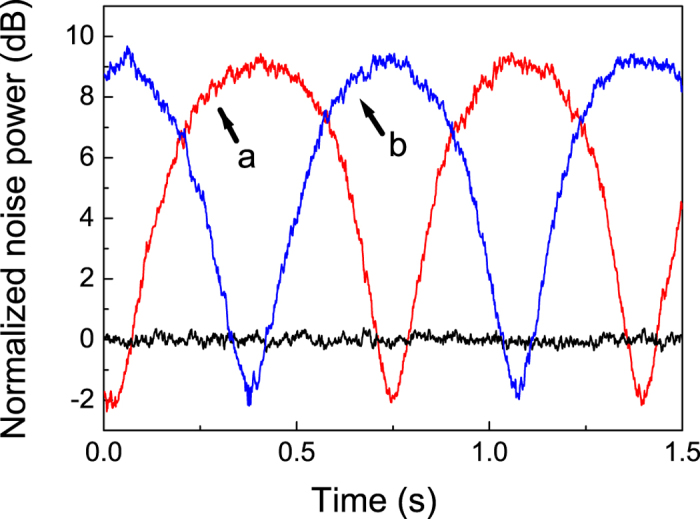
Frequency spectra of (**a**) 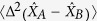 and (**b**) 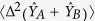 for our spatially separated entanglement at an analysis frequency of 10 MHz. The resolution of the spectrum analyzer is 300 kHz, and its video bandwidth is 100 Hz.

To investigate the entanglement correlation between Alice and Bob, they first are spatially separated by a distance of 20 cm. Note that this distance is much longer than the spatial pulse length of 2 mm that limits the minimum time needed to switch measuring quadrature amplitudes. Before interrogating the EPR correlation in the time-domain, we first checked the entanglement in the frequency domain. The subtraction and addition of the resulting photocurrents from the two sets of homodyne systems is recorded using a spectrum analyzer. The measured spectra for 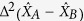 and 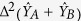 at a fixed analysis frequency of 10 MHz are shown in Fig. 3. The gains between two homodyne detections are set to unity. The traces are the normalized noise power of the sum and difference of two sets of HDs when the phase of the LO_*A*_ at Alice is fixed on measurement of one quadrature and the phase of LO_*B*_ at Bob is simultaneously scanned. (The blue trace is the amplitude quadrature sum, and the red trace is the phase quadrature difference.) When the two squeezed states are blocked, the HDs record the shot noise level, which is 7.5 dB above the amplifier noise. The sum and difference noise curves successively drop below the SNL. Taking the electronic noise of the HDs into account, a noise reduction of 2.0 ± 0.2 dB below the shot noise level for two beams is observed when the same quadrature is measured at both observer stations. However, that accounts for neither the detection efficiency nor propagation efficiency. The noise reduction is translated into correlation variance to get 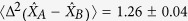 and 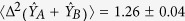. These measured values for the correlation variance are substituted into Eq. (2) and are given 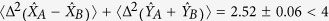, satisfying the inseparability criterion. This result attests to the quantum entanglement of the pulsed state generated by combining two squeezed vacua with a beam splitter.

**Figure 4:**
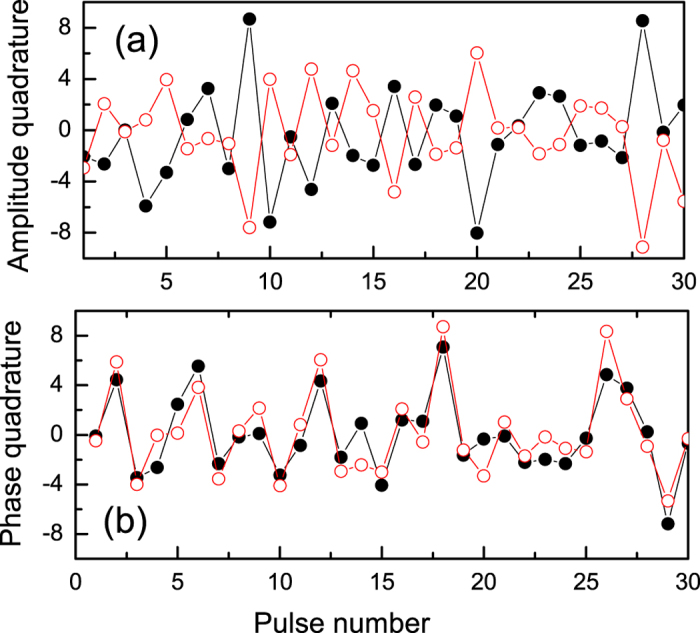
Measured correlations for 30 pulses. (**a**) Amplitude quadrature; (**b**) Phase quadrature.

**Figure 5:**
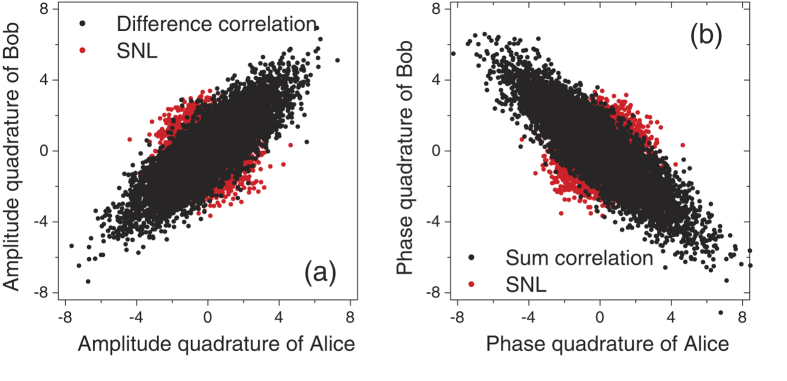
Measured correlation diagrams for 7600 pulses. (**a**) Amplitude quadrature; (**b**) Phase quadrature.

In the time-domain measurement, the quadrature values measured by Alice and Bob can be compared explicitly, in contrast to the variances of 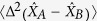 and 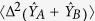 in the frequency domain. And the results directly demonstrate EPR correlation. To accomplish it, the outputs of the HD at both Alice and Bob are sampled by a digital oscilloscope. Once again, each quadrature value is obtained by integrating the sampled HD output over the time interval between successive pulses, i.e., the starting integration time “T” is chosen to obtain zero correlation coefficient. This method differs from that of previous works^11^,^25^ using continuous wave light sources where the time-resolved analysis was only performed in a limited time windows or the Fourier transform was only calculated at one fixed frequency. This is also different from the previous pulsed scheme in which only one quadrature of each pulse was sampled^20^,^21^. The quadrature values of 7600 pulses are obtained by numerically integrating a time sequence of 500,000 points, limited only by the storage size of the oscilloscope. The following results are directly obtained from these individual pulse measurements. In the time-domain analysis, the amplifier noise is not subtracted off. Figure 4 shows a typical example of the measured quadrature values, in which only 30 pulses are shown. The partial correlation and anti-correlation in the measured quadrature values of the entangled beams is evident, manifesting the originally devised correlations discussed by EPR. Figure 5 gives a direct representation of the correlation between the measured quadrature values for 7600 pulses of entangled beams and vacuum beams. Each point corresponds to one simultaneous measurement at Alice and Bob. As mentioned above, the measured values of entangled beams behaves in such a way that amplitude and phase quadratures are correlated and anti-correlated, respectively. We can directly calculate all elements of covariance matrix using the same data shown in Fig. 5.


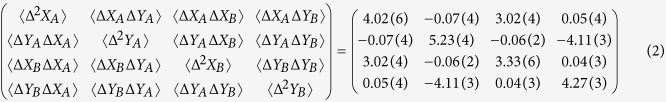


Positive and negative covariances represent correlation and anticorrelation, respectively. The correlation variance is 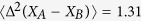 for amplitude quadrature and 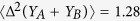 for phase quadrature, without taking into account of the detection dark noise, detection efficiency, and propagation efficiency. As a result, they are slightly smaller than the measured values in the frequency domain. The sufficient criterion for quantum entanglement holds according to 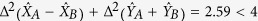. Therefore the generated state is also entangled in the temporal mode.

### Long distance separation

**Figure 6:**
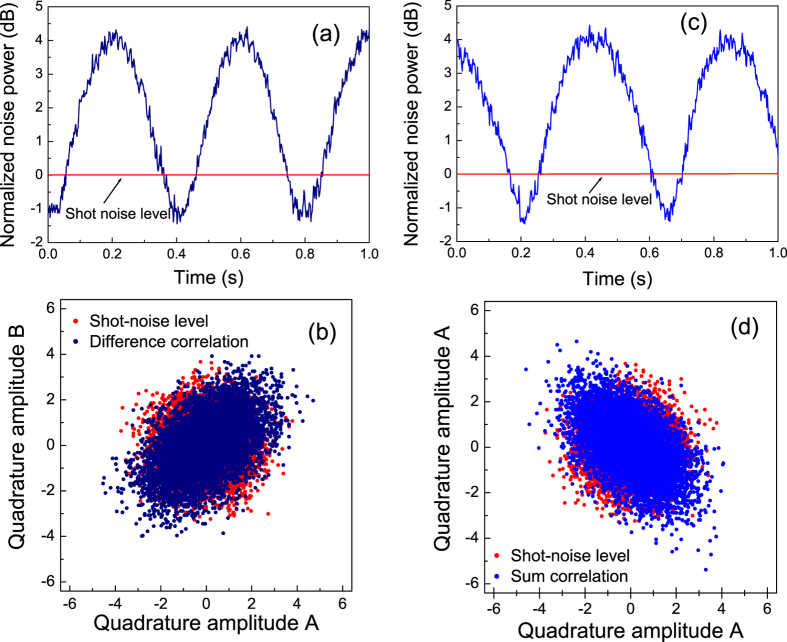
Measured entanglement correlation when Alice and Bob are spatially separated by a distance of 4.5 m. (**a**) Frequency spectrum of 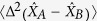 and (**b**) the corresponding correlation diagram for 7600 pulses in the time domain; (**c**) Frequency spectrum of 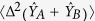 and (**d**) the corresponding correlation diagram for 7600 pulses in the time domain. In the frequency domain, the analysis frequency is 10 MHz, the resolution of spectrum analyzer is 300 kHz, and its video bandwidth is 100 Hz.

Figure 6 shows the measured entanglement correlation in both the frequency domain and the time domain when Alice and Bob are spatially separated at a distance of 4.5 m. The pump power of OPAs were halved compared to the short-distance experiment in order to reduce the anti-squeezing noise levels. The sum and difference noise curves successively drop below the SNL. Taking the electronic noise of the HDs into account, the noise reduction is 1.2 ± 0.2 dB and 1.3 ± 0.2 dB below the SNL for amplitude and phase quadratures, respectively. We translate the noise reduction into correlation variance and get of 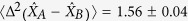 and 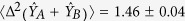. These measured values for the correlation variance substituted into Eq. (2) and give 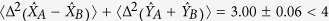. In the time domain, the covariance matrix is found to be


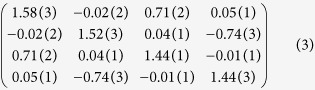


using the afore-mentioned method. The correlation variance is 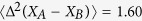 for the amplitude quadrature and 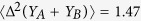 for the phase quadrature. Thus the criterion for quantum entanglement holds 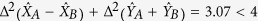, so that the generated state is also entangled in both the spectral domain and in the temporal domain, even when spatially separated by 4.5 m, which suffices to demonstrate the causal separation criterion. Compared to the correlation variance measured at a separation distance of 20 cm, the degradation may be due to the reduced spatial-mode matching efficiency caused by a longer propagation distance and additional mechanical vibrations, given that the measurements were carried out on two different optical benches.

## Discussion

These experiments constitute the first demonstration of entanglement with continuous variable that is sufficiently separated to verify the casual separation criterion. Such separation has been extensively discussed in the literature on entanglement with discrete variable and Bell’s inequalities. For two spatially separated subsystems at A and B, the causal separation criterion is specified by^2^


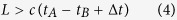


where *L* is the separation distance, *c* is the speed of light, *t*_*A*_ and *t*_*B*_ are the times of light from the source to positions A and B, and Δ*t* is the independent measurement time interval. In experiment, it is not difficult to take equal distances from source to A and B; this means that *t*_*A*_ = *t*_*B*_. Hence, the key of demonstrating causal separation is to separate two measurement stations at a distance of large enough than the *c*Δ*t*. For experiments using a continuous wave light source, the independent measurement time interval Δ*t* is relative not only to the measurement time (the bandwidth of the photodide) but also to the light coherence length. Hence, it is difficult to determine the measurement time interval for which successive measurements are independent and also measurements at different sites are correlated for continuous wave light. Fortunately, it is possible to limit it to lie within only the pulse duration when a pulsed laser is employed as the light source. In the worst case, the measurement time interval can be limited within the interval between successive pulses. The former result has shown that the measurement time interval can be made less than 13 ns. Hence, if two observers Alice and Bob are spatially separated by more than 4 m, the causal separation criterion will be achieved.

To strictly demonstrate EPR, the conditional variance between observers Alice and Bob should be such that measurement of either observable by Alice permits the inference of the same variable by Bob to better than the standard quantum limit. That is, the variance should satisfy the EPR criterion of 
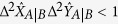
11,26. A value of 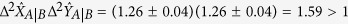 is measured when Alice and Bob are spatially separated by a distance of 20 centimeter. This result can be further promoted by an improvement of squeezing of the amplitude squeezed pulse trains or by a reduction of antisqueezing noise of them^27^.

In summary, we have demonstrated a spatially separated entanglement. It was characterized in terms of the inseparability criterion with an optimum result of 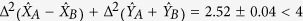 in the frequency domain and 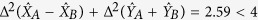 in the time domain when Alice and Bob are separated by a distance of 20 cm. The quantum correlation has also been observed when the two measurement stations are separated by a sufficient physical distance of 4.5 m. The sufficiently separated entanglement, together with the time resolved homodyne detection, provides the necessary resources for future realization of spatially-separated EPR paradox using continuous variables. Also it provides a new resource for future quantum information experiments such as demonstration of EPR inequality by conditional measurements.

## Additional Information

**How to cite this article**: Zhang, Y. *et al.* Experimental realization of spatially separated entanglement with continuous variables using laser pulse trains. *Sci. Rep.* **5**, 13029; doi: 10.1038/srep13029 (2015).
